# NCDs in low and middle-income countries - assessing the capacity of health systems to respond to population needs

**DOI:** 10.1186/1471-2458-14-S2-S1

**Published:** 2014-06-20

**Authors:** Pascale Allotey, Tamzyn Davey, Daniel D Reidpath

**Affiliations:** 1 Global Public Health, School of Medicine and Health Sciences, Monash University Malaysia, Sunway, Malaysia; 2 South East Asia Community Observatory (SEACO), Monash University, Segamat, Malaysia

## 

Health systems consist of individuals, organisations and process. They require leadership and governance to determine policy and direction and the resources to manage the needs of the populations they serve. They require evidence to guide the best health policies and programs, and ongoing surveillance to monitor the performance. They also require mechanisms for engaging with communities, not only to remain responsive to health needs, but also to facilitate the ability of communities to mobilise resources and to participate actively in promoting and managing their health. The role of communities as an integral part of health systems is increasingly important within the context of the growing chronic non-communicable diseases (NCDs) burden.

NCDs, namely cardiovascular disease, diabetes, cancer, and chronic respiratory diseases account for more morbidity and mortality globally than all other causes combined[1]. While the problem of NCDs continues to increase in all regions of the world, it disproportionally affects low and middle-income countries (LMICs), where approximately 80% of the global deaths from NCDs occur[1]. There are several reasons for this. First, many LMICs are still burdened with the prevention and management of communicable diseases [1]. This is compounded by the fact that health competes with a host of other priorities related to development including alleviation of poverty, access to education, gender equality, development of infrastructure, and mitigating against degradation of the environment [2]. Second, the wider environmental, political, social, and economic contexts in many LMICs are often not conducive to the health promoting behaviours[1-4]. Third, health systems in LMICs often lack adequate funding and were not designed to manage chronic conditions, with the financial burden of health care costs falling on the individuals, families, communities, and ultimately impacting the economy[5].

Current models of health service utilisation rely on repeated serial visits: designed for acute conditions, but financially unsustainable for chronic disease management [5]. Given the higher demand for ongoing care required for chronic disease management, community organisations and systems are uniquely placed to support health systems institutions and respond quickly to needs within communities.

Over the past 50 years, there has been an increased understanding of the importance of actively involving communities in health care and the potential of communities to successfully direct health interventions. The Alm Ata Declaration on Primary Health Care signed in 1978 enshrined the principle of working with communities for the first time in international health policy. The Declaration marked a change in roles and responsibilities in health by stating clearly that: “primary health care requires and promotes maximum community and individual self-reliance and participation in the planning, organization, operation and control of primary health care, making fullest use of local, national and other available resources; and to this end develops through appropriate education the ability of communities to participate”[6].

More recently, the theme of greater integration and participation with communities has been further formalised through the World Health Organization’s definition of the intrinsic goals of health systems[7,8]. There are three intrinsic goals that relate to the provision of good health, responsiveness to the expectations of the population and a fairness of financial contribution to mitigate against impoverishment. Working with communities is under the rubric of the second goal: to meet the legitimate universal expectations of the population. Evidence indicates that improvements in health system responsiveness has a positive effect on health outcomes through increased treatment compliance, relevant and timely provision of information to service providers, and continued use of health services – all of which makes health more likely[9]. People deem responsiveness to be important, and this, in part, accounts for why responsiveness relates to well-being. If health systems are responsive to communities’ expectations of how they wish to be treated, then their interactions with the system are more likely to improve their well-being [8,9].

The featured articles for this series arose primarily from selected papers presented at the first international conference on non-communicable diseases by the Nutrition Society of Malaysia. The theme was the engagement of multi-stakeholders and strategic partnerships in combating non-communicable diseases with several papers highlighting the growing role of communities. Further papers were solicited from low and middle income countries that built on critical issues raised by the initial presentations. The compilation of papers address national health systems in Ghana, China, and Malaysia and raise financing and health workforce concerns with regard to the escalating burden of NCDs in those countries. The papers draw out the significant challenges the systems face in the absence of stronger partnerships with communities. Xiao et al., in their article on a community-based approach to NCD management in China, point to shortages of qualified staff at the primary healthcare level. Similarly, de-Graft Aikins et al. describe the need for health workforce strengthening in Ghana to support universal health coverage. These concerns, common across countries at varying degrees of development, are likely to have serious implications for responsiveness. Limited resources and shortages of adequately trained health care providers, means responsiveness elements like choice of providers, prompt attention, and equitable spread of services, are less likely.

Mustapha et al. describe an integrative process of systems and community to address NCDs in Malaysia. The paper provides an example of an extensive system-wide response to NCDs in a high middle-income country context. The NCD prevention programme discussed involves wide-scale participation of and engagement with the community, with trained community members assisting with services like screening and health counselling. Notwithstanding the challenges acknowledged by the authors with regard to the sustainability of this intervention, such an approach is likely to facilitate the client orientation elements of responsiveness in terms of the population’s interactions with primary healthcare services. The extension of the intervention to remote and isolated communities not only indicates the achievement of equitable spread of responsiveness, but also makes responsiveness elements like prompt attention, and access to social support networks, more likely. Conversely, because of the extensive nature of the programme and limited resources, choice of provider is less likely, because non-medical providers are required to assist in maintaining the population-wide programme.

The other articles in this series point to particular sections of the population that are either vulnerable in terms of their NCD risk, or who are vulnerable in their own right (e.g. children). The protection of vulnerable groups in the population goes to the very core of knowing and responding to communities. Draper et al. and Norris et al. table the need to target adolescents and young adults for NCD risk-reduction interventions, especially with regard to type 2 diabetes mellitus and obesity. This group is important for three main reasons: 1.) they are in the phase of development when patterns of health behaviours for adulthood are established; 2.) in many LMIC, this age group constitutes a substantial proportion of the productive workforce; and 3.) the women in this group are in the pre-conception or conception phase of life, and their own health and health behaviours will impact their offspring. van Niekerk et al. highlight the challenges of protecting children in LMIC health settings and the importance of using evidence-based interventions and implementation science to ensure sustainability of interventions which are central to the health systems ability to protect and promote basic human rights. Finally, Jahan et al. discuss the value of community based research platforms to provide the evidence that supports the health systems ability to contextualise and prioritise services based on regular monitoring of the population demographics.

## Conclusions

The escalating burden of non-communicable diseases (NCDs) in low and middle-income countries (LMICs) is threatening the capacity of health systems to respond adequately to the populations they serve. This series features articles from LMICs in Africa, Asia, and Southeast Asia. The articles variously address the burden of NCDs in the LMIC context and the health system ability to respond to this burden given their specific communities. The collection highlights the challenge of maintaining universal health coverage in the face of rising NCDs; and the development and implementation of NCD interventions at the community level.

## Competing interests

The compilation of papers is supported through the CSR (corporate social responsibility) unit of the NovoNordisk Foundation based in Kuala Lumpur. None of the manuscripts relate to the promotion of any NovoNordisk products.

The authors have no competing interests.

## Authors' contributions

Allotey, Davey and Reidpath contributed to the drafting of this manuscript.

## Declarations

Funding for SEACO is provided by the Research Offices of Monash University campuses in Australia and Malaysia; the Faculty of Medicine, Nursing and Health Sciences; the Jeffrey Cheah School of Medicine and Health Sciences; and the Faculty of Arts.

This article has been published as part of *BMC Public Health* Volume 14 Supplement 2, 2014: Responsive health systems: working with the community on control of non-communicable diseases (NCDs). The full contents of the supplement are available online at http://www.biomedcentral.com/bmcpublichealth/supplements/14/S2.
